# Editorial Correspondence

**Published:** 1877-02

**Authors:** 


### EDITORIAL CORRESPONDENCE.

MACON, GA., January 28, 1877.

MR. EDITOR: It has never been my disposition or desire to detract anything from the reputation of a professional brother, especially one young in the cause, and energetic in investigation and research, and in writing this article I only wish to put myself on record, as having six or eight years ago first introduced to the notice of the profession the hypodermic administration of veratrum in all forms of convulsions, especially the hysterical and puerperal. My attention has just been called to the subject from reading an interesting article in the January number of your journal, written by J. W. Griggs, M.D., of West Point, Ga., on "the hypodermic use of the tincture veratrum veride in puerperal eclampsia," in which the Doctor claims priority for the mode of administering the medicine in this disease. It is indeed a most interesting and instructive subject, and I am satisfied of the originality of this idea in the mind of Dr. Griggs, and that he never saw or read my article on the subject. My views are contained in (at that time) an exhaustive paper read before the Georgia State Medical Association at its meeting held in Americus, Ga., in 1871. This same paper was published in the June number, 1871, of the *Richmond and Louisville Medical Journal*, and I think also in the May number of same year, of *Atlanta Medical and Surgical Journal.* On page 531 of above mentioned *Richmond and Louisville Journal*, I say: "I will now proceed to speak of veratrum veride, a most familiar remedy, and am sure there is no medicine in our materia medica that deserves more praise, and whose effects on the human system are more uniform, and consequently more to be relied upon. All are acquainted with its effects upon the human system when administered by the mouth, and it is therefore to its hypodermic use I invite your attention. * * * * * When arterial excitement or tumultuous action of the heart is sought to be controlled, this medicine *injected under the skin* in proper doses, accomplishes a desideratum most devoutly to be wished for. In the convulsions of children, the result of high arterial excitement, so trying to the physician and frightful to the relatives and friends, we possess a certain remedy in veratrum hypodermically administered. I then proceed to give its mode of injection, quantity and point of insertion; also speak of its combination with Squibb's compound liquor of opium. I also speak of its administration in pneumonia and all affections when rapid sedation is desired.

On page 632 of same journal, I state, "in puerperal convulsions veratrum is a most valuable remedy. I will give two recent cases in which I employed it, thereby better conveying to the mind the mode of its administration and its effects on the system." I then give the full history of a case dated "January 8, 1871," and another one dated "March 5, 1871." This latter case the most interesting one that ever came under my notice, either in a practice of 25 years or in reading. I say further on page 635 of same journal, "I have never seen any reference to the hypodermic use of veratrum, and hope by this imperfect account of its employment to attract the attention of the profession, that its value and efficacy may be further developed and illustrated." These extracts are sufficient to establish the fact that so far as I know I was first to employ veratrum in the manner indicated. I am glad Dr. Griggs has given this interesting subject so much attention, and that he has given the profession the benefit of his experience and observation. I have continued its use since the publication of my papers, and my subsequent experience but strengthens the views therein expressed. I hope to hear again from Dr. G., as he further observes this mode of treating convulsions. I have used veratrum combined with some of the preparations of opium in the distressing paroxysms of asthma with the happiest effect; always giving relief a few moments after being introduced under the skin. The doses of veratrum can only be arrived at by experience, with each particular case, always beginning with a minimum dose and gradually increasing it; great caution being used. If an over-dose is administered, it can readily, promptly and certainly be counteracted by the injection of five or ten minimums of a solution of sulphate of atropine in the proportion of one grain to one ounce of water.

Yours very truly, WM. A. GREENE.

---

BOSTON, GA., Jan. 22, 1877.

*Editors Atlanta Medical and Surgical Journal:*

A cure for opium eating is certainly desirable just now, and I therefore contribute the following:

I was called to see Mrs. M. S., aged 40 years, and the mother of five children. The history of her case disclosed the fact that about six years ago, when pregnant with her last child or children—for she gave birth to twins—she suffered excruciating pain in the region of the uterus and ovaries, for which her attending physician prescribed opium. She continued the use of it until her delivery.

Having become so accustomed to the use of it, she continued the habit after the pain had entirely subsided. She thought of no difficulty in suspending at any time, not dreaming that she was enslaving herself to so baneful a habit, from which it is no easy matter to be freed. My first visit was made 30th of October last, at which time she informed me that she was in the habit of taking about forty grains of morphine daily. I found her nervous system prostrated—not being able for any length of time to stay in bed, to set up, or stand on her feet; and attended with the usual lassitude and depression of spirits, wanting to die, etc. Having secured the co-operation of her husband and friends, I promised to take her case and do the best I could.

I had desired an opportunity to try what I believed would effect a permanent cure, and commenced by giving her one grain of the extract nux vomica every six hours, and the hypodermic use of three grains of morphine morning and evening. I continued this for five days, my patient suffering considerably, but not affected by the nux vomica. On the sixth day I increased the dose of nux vomica to one grain every four hours, and decreased the morphine to two and a half grains, and continued this for five days more; at the end of which time, my patient still not being affected by the nux vomica, I concluded to try how much she could bear, and ordered her to take three grains every two hours until she came fully under the influence of the drug. I was sent for in great haste in about fourteen hours, and found my patient unable to walk, on account of great nervous tension, but no other bad effects. I ordered her thirty grains of chloral hydrate, which soon relieved her, and she was able to walk, and said she felt better than she had since the treatment began. I ordered the nux vomica to be again taken in the same dose and time. She took nine more grains in six hours, which made thirty grains in the twenty-four hours. On the next day she again took thirty grains; on the next day took twenty-eight grains; on the next day took thirty grains; on the next day she could take but twenty-four grains. From this time the dose had to be gradually decreased, until the 30th December, when she was taking but one grain, at which time I ordered the nux vomica to be discontinued.

It has now (January 22d) been three weeks since she took any of the nux vomica, and more than sixty days since taking any of the opium, and says she feels as well as she ever did in her life. Has good appetite, rests well at night, and in every way looks and feels like a new person, without any desire to again use the drug.

In this case I desire to direct particular attention to the great amount of strychnia the animal economy can bear, and its beneficial effects in such cases; for if it will do in this case and cure, what may we not expect from it in cases very nearly similar, as chronic alcoholism, etc. I have given it to men suffering from the effects of a debauch, and find it far preferable to bromide of potassa, hydrate of chloral, valerian, and other nervous sedatives that are generally given. There can be no doubt of the good quality of the nux vomica used in this case, for I took two grains of it myself, and it affected me very sensibly.

W. A. Pugh, M.D.

*Drs. J. G. and R. W. Westmoreland, Editors:*

I desire to contribute to the cause of humanity by giving my course in the treatment of twelve inveterate opium eaters. I think I am safe in saying that this *alarming* and *growing* habit is amenable to scientific, rational treatment, at least, my experience warrants me in believing so.

I have often in my professional career attempted to relieve this class of unfortunates by the daily reduction of their dose. Sometimes I inform them—reduce their dose in their presence—then again I take advantage of a little secrecy, and for a short time they do well, but *invariably* they ultimately quit the treatment, and pronounce it a humbug. I suppose this is the experience of others.

If patients can be relieved it is by the gradual reduction plan. Yet we who have tried it know it is difficult to carry out. We know there is success in it, but how to get to it is the question.

My plan is quite different from Collins, Beck & Co., yet I believe that our remedy is the same. As to color of hair, eyes, skin, etc., I care nothing; as we all know this is *bumcomb.* All I want to know is the exact dose and how often taken, and I generally enquire as to impairment of digestion and constipation, as it is necessary to the successful management of a case to overcome these difficulties.

If I find a patient using six grains per day, I give the following preparation :

| | |
|---|---|
| Morphine . . . . . . . . . | 64 gr. |
| Sulph. Strichnine . . . . . . | 1 gr. |
| Water . . . . . . . . . . | 2 oz. |
| Simple Syrp. . . . . . . . . | 6 oz. |

Dose—teaspoonful three times a day, not to be increased except by my advice. You see from this formula that I reduce from six grs. per day, down to four. You will see, allowing six teaspoonfuls to the oz. the preparation will last sixteen days. I find this time about long enough to run on this prescription. Now should I find this formula to work well, and it always will if you can get them through the first sixteen days, I reduce again—will give the following :

| | |
|---|---|
| Morphine . . . . . . . . | 56 gr. |
| Strichnine . . . . . . . . . | 1 gr. |

| | | |
|---|---|---|
| Water | . . . . . . . . | 2 oz. |
| Simple Syrp. | . . . . . . . | 6 oz. |

Dose—same as before.

You will see I reduce from 4 grs. per day down to $3\frac{1}{2}$ and so on. I never reduce the strichnine, for all know it has a good effect in constipation dependent upon deficient tone of the muscular coat of the large bowels, in addition to its taking to some extent the place of opium. I have by this plan of gradual reduction run them perfectly out, and finally only give them the strichnine. I assure you that in order to meet with almost universal success, we have only to exercise a little management and to draw heavily upon the imagination, which we are justified in doing.

I find it necessary to convince them at the beginning that the object can be gained, and I never allow them to doubt its ultimate accomplishment. The imagination plays a very important part in the treatment of these poor miserables, and we should not hesitate to take advantage of this when the destiny of a human being is involved.

Very respectfully, W. C. Blalock, M.D.,
Barnesville, Ga.

